# Revisiting the genotypes of *Theileria equi* based on the V4 hypervariable region of the 18S rRNA gene

**DOI:** 10.3389/fvets.2024.1303090

**Published:** 2024-03-15

**Authors:** Anil Kumar Nehra, Ansu Kumari, Aman Dev Moudgil, Sukhdeep Vohra

**Affiliations:** ^1^Department of Veterinary Parasitology, Lala Lajpat Rai University of Veterinary and Animal Sciences, Hisar, Haryana, India; ^2^Department of Veterinary Medicine, Lala Lajpat Rai University of Veterinary and Animal Sciences, Hisar, Haryana, India

**Keywords:** *Theileria equi*, phylogeny, genetic characterization, genotypes, clades

## Abstract

**Introduction:**

Equine theileriosis, an economically important disease that affects horses and other equids worldwide, is caused by a tick-borne intracellular apicomplexan protozoa *Theileria equi*. Genotyping of *T. equi* based on the 18S rRNA gene revealed the presence of two, three, four or five genotypes. In previous published reports, these genotypes have been labelled either alphabetically or numerically, and there is no uniformity in naming of these genotypes. The present study was aimed to revisit the phylogeny, genetic diversity and geographical distribution of *T. equi* based on the nucleotide sequences of the V4 hypervariable region of the 18S rRNA gene available in the nucleotide databases.

**Methods:**

Out of 14792 nucleotide sequences of *T. equi* available in the GenBank™, only 736 sequences of *T. equi* containing the complete V4 hypervariable region of the 18S rRNA gene (>207 bp) were used in multiple sequence alignment. Subsequently, a maximum likelihood phylogenetic tree was constructed based on the Kimura 2-parameter model (K2+I).

**Results:**

The phylogenetic tree placed all the sequences into four distinct clades with high bootstrap values which were designated as *T. equi* clades/ genotypes A, B, C and D. Our results indicated that the genotype B of Nagore et al. and genotype E of Qablan et al. together formed the clade B with a high bootstrap value (95%). Furthermore, all the genotypes probably originated from clade B, which was the most dominant genotype (52.85%) followed by clades A (27.58%), and C (9.78%) and D (9.78%). Genotype C manifested a comparatively higher genetic diversity (91.0-100% identity) followed by genotypes A (93.2-99.5%), and B and D (95.7-100%). The alignment report of the consensus nucleotide sequences of the V4 hypervariable region of the 18S rRNA gene of four *T. equi* genotypes (A-D) revealed significant variations in one region, between nucleotide positions 113-183, and 41 molecular signatures were recognized. As far as geographical distribution is concerned, genotypes A and C exhibited far-extending geographical distribution involving 31 and 13 countries of the Asian, African, European, North American and South American continents, respectively. On the contrary, the genotypes B and D exemplified limited distribution with confinement to 21 and 12 countries of Asian, African and European continents, respectively. Interestingly, genotypes A and C have been reported from only two continents, viz., North and South America. It was observed that genotypes A and C, and B and D exhibit similar geographical distribution.

**Discussion:**

The present study indicated the presence of only four previously described T. equi genotypes (A, B, C and D) after performing the molecular analyses of all available sequences of the complete V4 hypervariable region of the 18S rRNA gene of *T. equi* isolates in the GenBank™.

## 1 Introduction

Equine piroplasmosis (EP) consists of two tick-borne diseases, equine theileriosis and babesiosis, which are, respectively, caused by hemoprotozoa, *Theileria equi* and *Babesia caballi* (1, 2). Besides worldwide geographical distribution, EP is an economically important tick-borne disease with high morbidity and mortality rates (1, 3). *Theileria equi* and *B. caballi* are transmitted by ixodid ticks under natural conditions which act as biological vectors for them (4). Additionally, transplacental (5, 6), and iatrogenic transmission by the use of contaminated needles and syringes, surgical instruments, and blood transfusions have been reported (7). Both *T. equi* and *B. caballi* infections cause subclinical to acute diseases, and the clinical signs are usually similar and non-specific in nature (4, 8). In general, *T. equi* causes a more severe clinical disease compared to *B. caballi* (9).

Genotyping of *T. equi* based on 18S rRNA gene revealed the presence of two (10, 11), three (12–14), four (15–18) or five (19–27) genotypes. In previous published reports, these genotypes have been labelled either alphabetically (12, 14–17, 19–27) or numerically (13, 18), and there is no uniformity in naming of these genotypes. The genotyping has been reported from several countries namely, Brazil (13, 24, 28), Chile (18), China (25), Croatia (29), Israel and the Palestinian Authority (27), Italy (14, 17), Jordan (19, 21), Mongolia (20), South Africa (12), Spain (10, 26, 30), Sudan (15), Tunisia (31), Turkey (23), and the United States of America (16). Besides, individual studies are based on few limited isolates/ field samples and no qualified study has been done using all the sequences available in the nucleotide databases. With the addition and release of more sequences from separate geographical areas, a change in number of *T. equi* genotypes is expected (12, 21, 32). Here, we make use of an expanded number of rRNA sequences to better understand the phylogeny of *T. equi*. Additionally, we need to better understand the genetic diversity of *T. equi*. Therefore, we intended to revisit the phylogeny, genetic diversity and geographical distribution of *T. equi* genotypes based on the nucleotide sequences of the V4 hypervariable region of the 18S rRNA gene available in the nucleotide databases.

## 2 Materials and methods

### 2.1 Retrieval of nucleotide sequences

Out of 14,792 nucleotide sequences of *T. equi* available in the GenBank™, the 18S rRNA sequences of *T. equi* (*n* = 927) were downloaded in the FASTA format from the nucleotide database accessed in March, 2023. A dataset was created with 736 sequences of *T. equi* containing the complete V4 hypervariable region of the 18S rRNA gene (>207 bp) and the remaining sequences were discarded. The sequences derived from blood, spleen of the aborted foetus and ticks of various vertebrate and invertebrate hosts, *viz.*, horse, African wild donkey (*Equus africanus*), Asiatic wild ass (*Equus hemionus*), zebra (*Equus quagga*), *Equus ferus caballus*, *Rhipicephalus sanguineus* ticks of dogs, *Rhipicephalus sanguineus s.l.*, naturally infected dogs, German shepherd dog, *Rhipicephalus bursa*, *Canis lupus familiaris*, *Hyalomma excavatum*, *Hyalomma anatolicum*, Black rhinoceros (*Diceros bicornis*), *Rhipicephalus annulatus* ticks of cattle, domestic donkey (*Equus asinus*), *Haemaphysalis* sp., *Dermacentor nuttalli* isolated from horse, *Ixodes ricinus*, *Rhipicephalus appendiculatus* and *Rhipicephalus evertsi evertsi*, were included in the analysis. In addition, *Theileria haneyi* sequences containing the complete V4 hypervariable region of the 18S rRNA gene (*n* = 06) were also included in the analysis. The details of accession numbers used in the current study are provided in Supplementary Table S1.

### 2.2 Multiple sequence alignment and phylogenetic analyses based on 18S rRNA gene

The dataset containing 18S rRNA sequences was uploaded to MEGA-X software version 10.1.7 for sequence alignment (33). Sequences of varying lengths were aligned using ClustalW and their unequal lengths were trimmed at one or both ends for equalization using a reference sequence of the V4 hypervariable region of 18S rRNA gene of *T. equi* (Accession number MN818862). The nucleotide identities were computed using MegAlign (DNASTAR) software (34).

**Figure 1:**
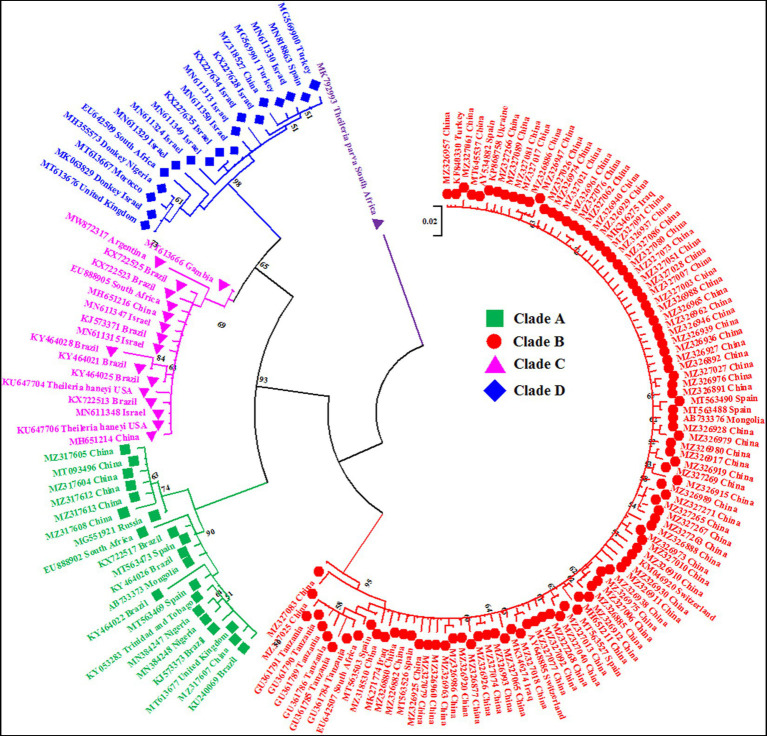
A circular maximum likelihood tree based on the V4 hypervariable region of the 18S rRNA gene clearly depicts the four genotypes/clades (A, B, C and D) of *T. equi* due to extensive nucleotide heterogeneity in this region. The taxon name of each sequence is depicted by its accession number followed by the country of origin. The color coding of different clades is as below: Genotype A-Green font color with green filled square as taxon marker; Genotype B-Red font color with red filled circles as taxon markers; Genotype C-Pink font color with pink filled triangles as taxon markers; Genotype D-Blue font color with blue filled rhombi as taxon markers; Outgroup-Purple font color with purple filled inverted triangle as taxon marker.

**Figure 2:**
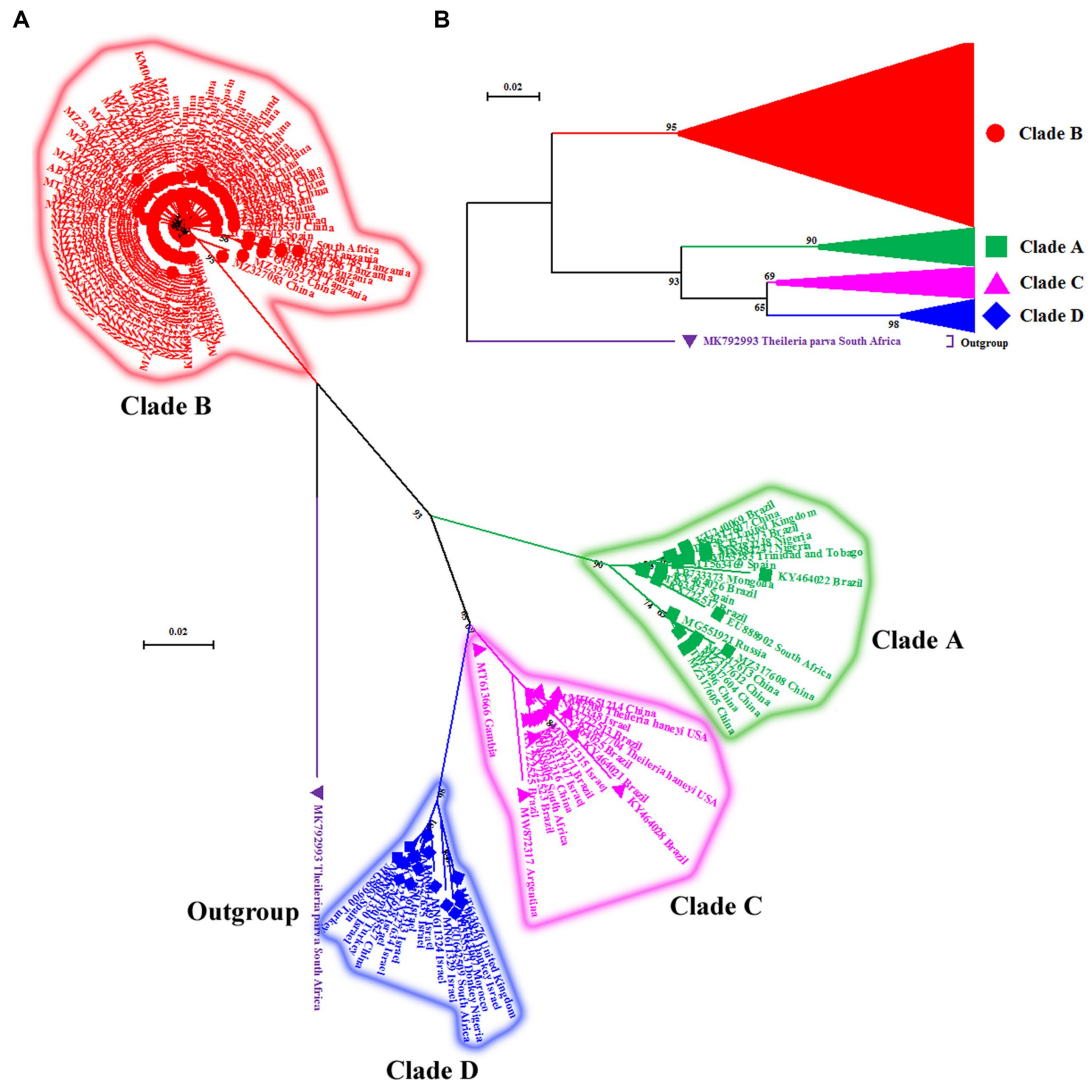
**(A)** Cladogram depicting clear distinction between four genotypes (A–D) of *T. equi* based on the V4 hypervariable region of the nuclear 18S rRNA gene. The taxon name of each sequence is depicted by its accession number followed by the country of origin. **(B)** The compressed tree depicting the phylogenetic relationship between four genotypes (A–D) of *T. equi*. It is evident that all genotypes have probably originated from clade B. The color coding of different clades is as below: Genotype A-Green font color with green filled square as taxon marker; Genotype B-Red font color with red filled circles as taxon markers; Genotype C-Pink font color with pink filled triangles as taxon markers; Genotype D-Blue font color with blue filled rhombi as taxon markers; Outgroup-Purple font color with purple filled inverted triangle as taxon marker.

For phylogenetic analysis, the V4 hypervariable region of the 18S rRNA gene sequences was subjected to Multiple Alignment using Fast Fourier Transform (MAFFT) online software (35). The aligned sequences were analyzed using MEGA-X software version 10.1.7 to predict the most suitable model based on Akaike and Bayesian information criterion. Subsequently, a maximum likelihood phylogenetic tree was constructed based on the Kimura 2-parameter model (K2 + I; 36) as previously described in detail by Nehra et al. (32). The rate variation model allowed some sites to be evolutionarily invariable ([+I], 26.67% sites). The final alignment involved 743 nucleotide sequences (736 *T. equi*, 06 *T. haneyi* and 01 outgroup) with a total of 213 positions. *Theileria parva* (MK792993, South Africa) was used as an outgroup species for rooting (33). The reliability of the tree was assessed by 1,000 bootstrap replications (Figures 1, 2).

For ease in display of results of the phylogenetic analysis, the identical sequences of *T. equi* were removed to obliterate the superfluous sequences; consequently, only 154 and six sequences of *T. equi* and *T. haneyi* were included, respectively. The phylogenetic analysis was again performed as described before to generate Figures 1, 2.

## 3 Results

### 3.1 Phylogenetic analyses

The maximum likelihood tree placed all the sequences into four distinct clades/genotypes with high bootstrap values which were designated as *T. equi* clades A, B, C, and D (Figures 1, 2A,B). All genotypes probably originated from clade B. The clades A, B, C, and D consisted 203, 389, 78, and 72 sequences originating from different vertebrate and invertebrate hosts, respectively (Table 1). The 78 sequences of Clade C encompassed 72 sequences of *T. equi* and six sequences of *T. haneyi*. This clearly indicated that the *T. equi* clade B is the most dominant genotype (52.85%) followed by the clades A (27.58%), and C (9.78%) and D (9.78%). However, the *T. equi* clades A, B, C, and D in Figures 1, 2A displayed only 21, 104, 17, and 18 sequences, respectively, due to the removal of superfluous sequences from the analysis.

**Table 1:** Geographical distribution of *T. equi* genotypes, along with the clade-wise and country-wise distributions of the partial 18S rRNA gene sequences (*n* = 736) involved in the analysis.

Group	Percentage	Continent	Country (Number of sequences)	Number of countries
Clade A (*n* = 203)	27.58%	Asia, Africa, Europe, North America and South America	Brazil (34); Chile (10); China (12); Colombia (02); Croatia (01); Cuba (02); Egypt (08); France (03); Gambia (03); India (01); Iran (02); Iraq (02); Israel (28); Italy (02); Jordan (02); Mongolia (02); Morocco (03); Nigeria (07); Paraguay (01); Portugal (10); Russia (04); Saudi Arabia (16); South Africa (07); South Korea (01); Spain (24); Sudan (01); Trinidad and Tobago (03); Tunisia (01); Turkey (02); United Kingdom (03); United States of America (06)	31
Clade B (*n* = 389)	52.85%	Asia, Africa and Europe	Austria (03); China (243); Hungary (10); Iraq (05); Ireland (06); Italy (02); Kazakhstan (02); Mongolia (07); Poland (03); Portugal (23); Russia (03); Saudi Arabia (02); South Africa (01); South Korea (02); Spain (56); Sudan (01); Switzerland (05); Tanzania (08); Turkey (01); Ukraine (03); United Kingdom (03)	21
Clade C (*n* = 72)	9.78%	Asia, Africa, Europe, North America and South America	Algeria (01); Argentina (01); Brazil (44); China (04); Cuba (01); Gambia (02); Israel (08); Italy (01); Nigeria (03); Saint Kitts and Nevis (01); South Africa (04); Tunisia (01); United States of America (01)	13
Clade D (*n* = 72)	9.78%	Asia, Africa and Europe	China (04); Israel (33); Morocco (03); Nigeria (06); Oman (01); South Africa (01); Spain (01); State of Palestine (04); Sudan (08); Tunisia (01); Turkey (09); United Kingdom (01)	12

### 3.2 Genetic diversity

The percent nucleotide identity of all the *T. equi* sequences was 91.0–100%. Nucleotide variations were ascertained in the V4 hypervariable region of the 18S rRNA gene at isolated places upon multiple sequence alignment. The careful examination unraveled four disparate genotypes, *viz.*, *T. equi* clades A, B, C, and D.

**Figure 3:**
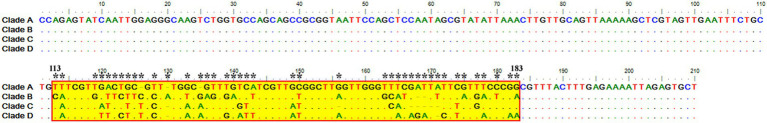
Multiple sequence alignment of the consensus sequences of the V4 hypervariable region of the 18S rRNA gene of *T. equi* genotypes A, B, C, and D exhibited significant variations between nucleotide positions 113–183 (shaded yellow in red box). A total of 41 molecular signature residues (marked *) were identified in the V4 hypervariable region.

Sequence analysis of the consensus V4 hypervariable region sequences of *T. equi* genotypes A, B, C, and D revealed significant nucleotide variations between positions 113–183 and identified 41 molecular signatures. Moreover, no nucleotide variations were observed at other places among clades (Figure 3).

#### 3.2.1 *Theileria equi* genotype A

**Figure 4:**
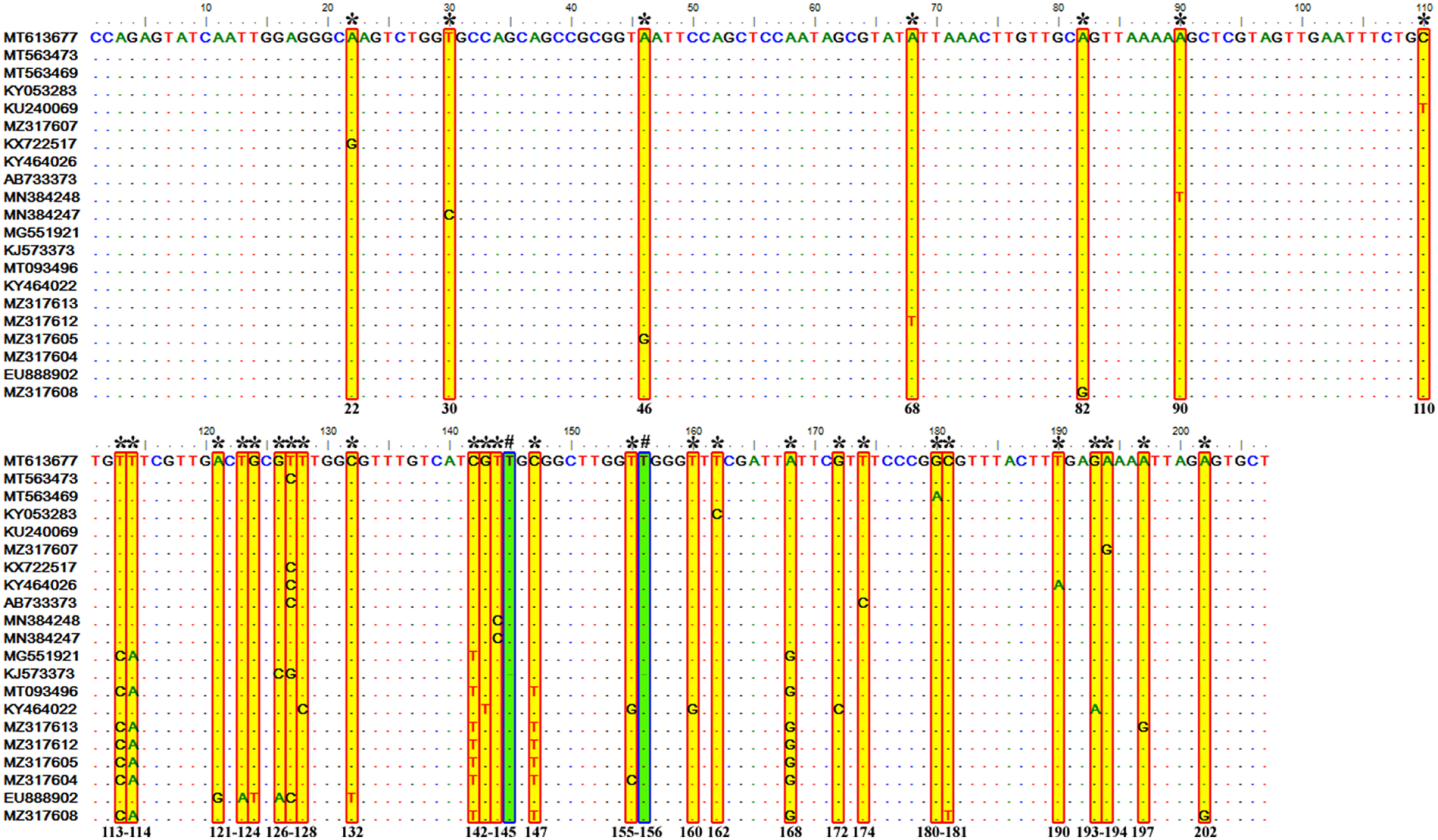
Sequence variations detected in the V4 hypervariable region of the 18S rRNA gene of *T. equi* genotype A upon multiple sequence alignment. The identical sequences were removed from the alignment. Single nucleotide substitutions were documented at 33 places within this genotype (marked * and shaded yellow in red box). Similarly, single nucleotide deletions (marked # and shaded green in blue box) were observed at two positions (145 and 156).

It consisted of 203 sequences of *T. equi* which displayed 93.2–99.5% similarity amongst each other (Tables 1, 2). Additionally, it exhibited 85.5–89.9%, 85.0–94.7%, and 83.6–89.9% sequence similarity with *T. equi* genotypes B, C, and D, respectively (Table 3). The high similarity (85.0–94.7%) with genotype C suggested its close association with this genotype, which is also displayed in the phylogenetic analysis (Figures 1, 2). Single nucleotide substitutions and deletions at 33 and two positions (145 and 156), respectively, were documented in the V4 hypervariable region of this genotype upon sequence analysis (Figure 4). Likewise, single nucleotide variations were observed at 116, 62, and 64 places when compared with *T. equi* genotypes B, C, and D, respectively.

**Table 2:** A breakdown of the percent nucleotide identity of *T. equi* genotypes based on the V4 hypervariable region of the 18S rRNA gene.

Group	Designation	Percent identity within genotype	Closest *T. equi* genotype	Percent identity with closest *T. equi* genotype
Clade A	A	93.2–99.5	C	85.0–94.7
Clade B	B	95.7–100	A	85.5–89.9
Clade C	C	91.0–100	D	88.2–95.1
Clade D	D	95.7–100	C	88.2–95.1

**Table 3:** Percent nucleotide identity matrix of *T. equi* genotypes based on the V4 hypervariable region of the partial 18S rRNA gene.

Genotype	A	B	C	D
A	–	85.5–89.9	85.0–94.7	83.6–89.9
B	85.5–89.9	–	79.5–86.5	84.6–88.9
C	85.0–94.7	79.5–86.5	–	88.2–95.1
D	83.6–89.9	84.6–88.9	88.2–95.1	-

#### 3.2.2 *Theileria equi* genotype B

It contained 389 sequences of *T. equi* which manifested 95.7–100% nucleotide homology (Table 2). It displayed 85.5–89.9%, 79.5–86.5%, and 84.6–88.9% nucleotide identity with *T. equi* genotypes A, C, and D, respectively (Table 3); consequently, evinced its close association with genotype A (85.5–89.9%). The multiple sequence alignment report of the V4 hypervariable region of the 18S rRNA gene revealed nucleotide variations at 96 places within this genotype. Similarly, it showed sequence variations at 116, 122 and 118 places when compared with the 18S rRNA sequences of *T. equi* genotypes A, C and D, respectively.

#### 3.2.3 *Theileria equi* genotype C

**Figure 5:**
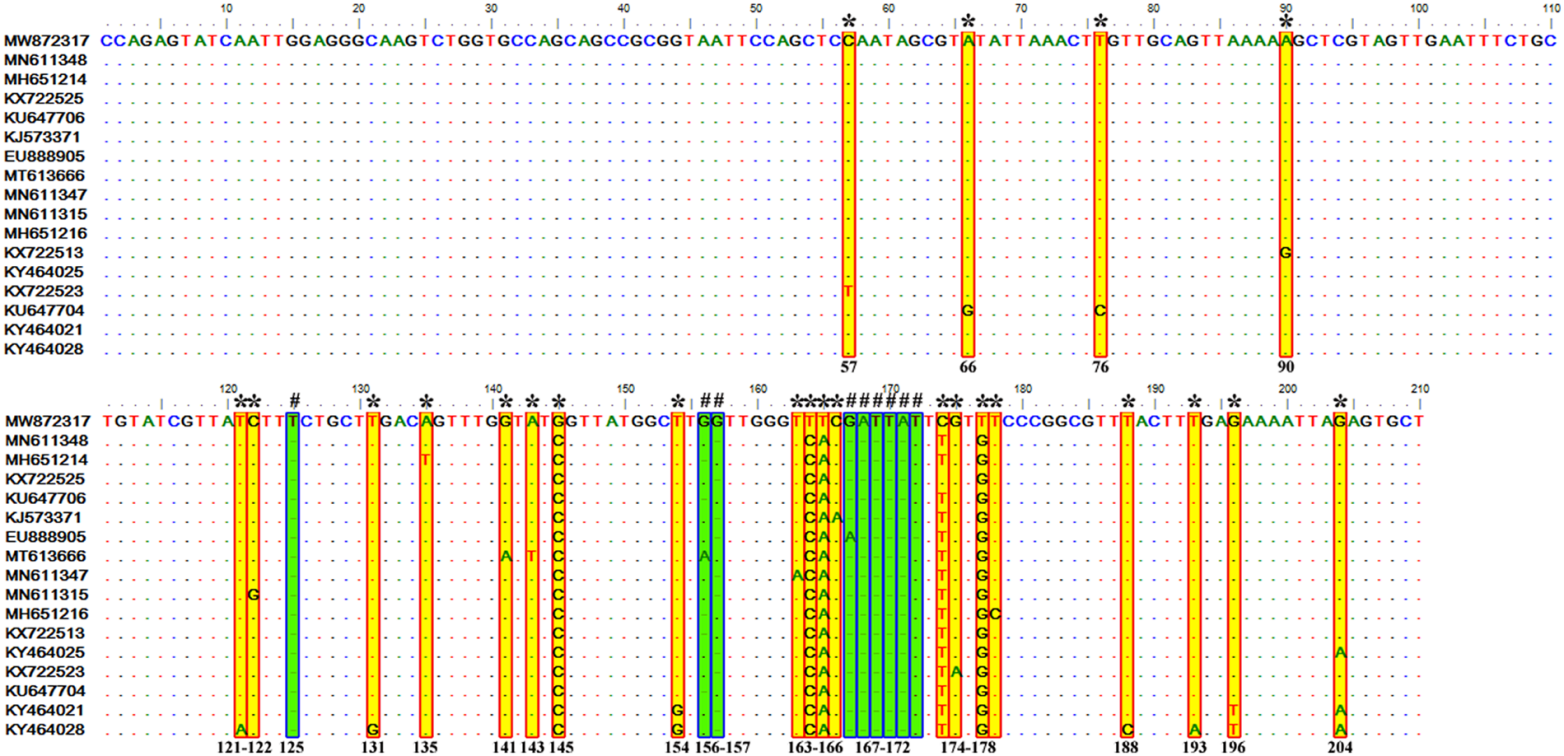
Sequence variations detected in the V4 hypervariable region of the 18S rRNA gene of *T. equi* genotype C upon multiple sequence alignment. The identical sequences were removed from the analysis. The alignment report of this genotype exhibited single nucleotide substitution (marked * and shaded yellow in red box) and deletion (marked # and shaded green in blue box) at 24 and nine places (125, 156, 157, 167, 168, 169, 170, 171, and 172), respectively.

It contained 72 sequences of *T. equi* which manifested 91.0–100% nucleotide homology (Table 2). Additionally, it showed 85.0–94.7%, 79.5–86.5%, and 88.2–95.1% nucleotide identity with *T. equi* genotypes A, B, and D, respectively (Table 3). Therefore, it indicated its close association with genotype D (88.2–95.1%) which is also displayed in the phylogenetic analysis (Figures 1, 2). The sequence analysis of this genotype demonstrated single nucleotide substitutions and deletions at 24 and nine places (125, 156, 157, 167, 168, 169, 170, 171, and 172), respectively (Figure 5). Likewise, single nucleotide variations were observed at 62, 122, and 50 places when compared with *T. equi* genotypes A, B, and D, respectively.

#### 3.2.4 *Theileria equi* genotype D

**Figure 6:**
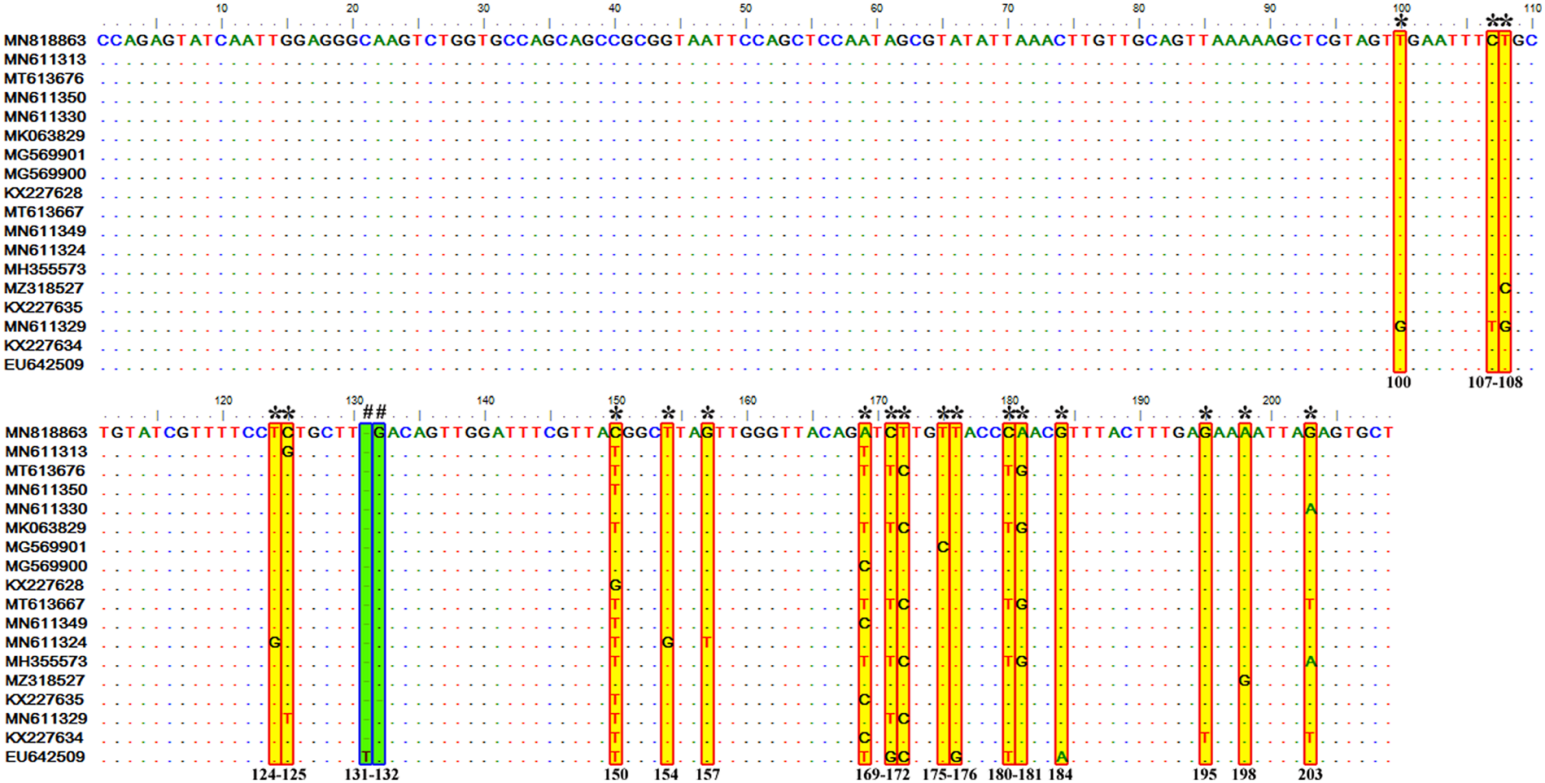
Sequence variations detected in the V4 hypervariable region of the 18S rRNA gene of *T. equi* genotype D upon multiple sequence alignment. The identical sequences were removed from the analysis. The alignment report of this genotype exhibited single nucleotide substitution (marked * and shaded yellow in red box) and deletion (marked # and shaded green in blue box) at 19 and two places (131 and 132), respectively.

Similar to the genotype C, it contained 72 sequences of *T. equi* which manifested 95.7–100% nucleotide homology (Table 2). Furthermore, it exhibited 83.6–89.9%, 84.6–88.9%, and 88.2–95.1% nucleotide identity with *T. equi* genotypes A, B, and C, respectively (Table 3). Thus, it suggested its close association with genotype C (88.2–95.1%) which is also displayed in the phylogenetic analysis (Figures 1, 2). The sequence analysis of this genotype demonstrated single nucleotide substitutions and deletions at 19 and two places (131 and 132), respectively (Figure 6). Likewise, single nucleotide variations were documented at 64, 118, and 50 places when compared with *T. equi* genotypes A, B, and C, respectively.

### 3.3 Geographical distribution

**Figure 7:**
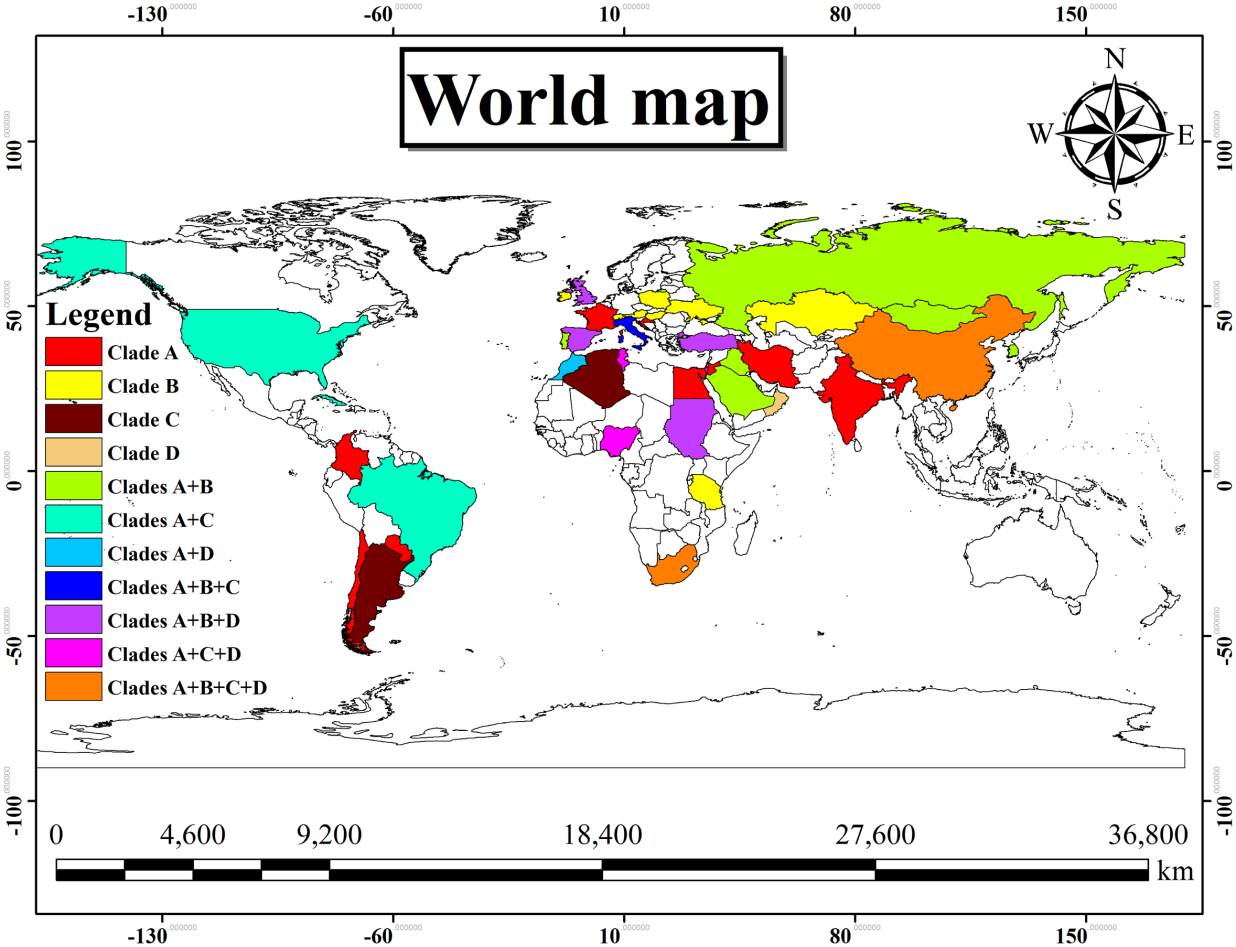
Geographical distribution of the different genotypes (A–D) of *T. equi* exemplified a very wide distribution involving Asian, African, European, North and South American continents. The color illustrations are depicted in the legend of this figure. It was observed that the genotypes A and C, and B and D exhibit similar geographical distribution.

In Table 4, the country-wise distribution of various genotypes of *T. equi* is enlisted. *Theileria equi* genotype A exhibited the most widespread and far-extending geographical distribution involving 31 countries of the Asian, African, European, North American and South American continents. Similar to this genotype, genotype C extended its distribution to 13 countries of the Asian, African, European, North American and South American continents. On the contrary, the genotypes B and D exemplified limited distribution with confinement to 21 and 12 countries of Asian, African, and European continents, respectively (Table 1). Genotypes A and C are the only genotypes recorded from five continents, *viz.*, Asia, Africa, Europe, North America, and South America. Furthermore, all the four genotypes (A-D) have been reported from three continents namely, Asia, Africa, and Europe. Interestingly, genotypes A and C have been reported from only two continents, *viz.*, North and South America. It was observed that genotypes A and C, and B and D exhibit similar geographical distribution. Amongst all the *T. equi* genotypes, genotype D revealed the most restricted distribution with confinement to 12 countries only (Table 1). The genotype C showed relatively wide-stretched distribution compared to the genotype D with reports from 13 countries (Figure 7). One (A/B/C/D), two (A + B/A + C/A + D), three (A + B + C/A + B + D/A + C + D) and four (A + B + C + D) genotypes have been reported from 23, 11, seven, and two countries, respectively. China and South Africa are thus far the only countries harboring all the four genotypes of *T. equi* (Table 4).

**Table 4:** Country-wise breakdown of the *T. equi* genotypes based on the V4 hypervariable region of the 18S rRNA gene.

Country	Genotype
Algeria	C
Argentina	C
Austria	B
Brazil	A and C
Chile	A
China	A, B, C, and D
Colombia	A
Croatia	A
Cuba	A and C
Egypt	A
France	A
Gambia	A and C
Hungary	B
India	A
Iran	A
Iraq	A and B
Ireland	B
Israel	A, C, and D
Italy	A, B, and C
Jordan	A
Kazakhstan	B
Mongolia	A and B
Morocco	A and D
Nigeria	A, C, and D
Oman	D
Paraguay	A
Poland	B
Portugal	A and B
Russia	A and B
Saint Kitts and Nevis	C
Saudi Arabia	A and B
South Africa	A, B, C, and D
South Korea	A and B
Spain	A, B, and D
State of Palestine	D
Sudan	A, B, and D
Switzerland	B
Tanzania	B
Trinidad and Tobago	A
Tunisia	A, C, and D
Turkey	A, B, and D
Ukraine	B
United Kingdom	A, B, and D
United States of America (USA)	A and C

## 4 Discussion

The current study aimed to investigate the genetic diversity of *T. equi* based on the V4 hypervariable region of the 18S rRNA gene, as it influences both the transmission of the disease and sensitivity of the diagnostic tests. Even though the equine merozoite antigen (*EMA*)-1 (20, 37–40) and β-tubulin (41) genes have been targeted, the hypervariable regions of the 18S rRNA gene are considered as the most suitable target for identification, phylogenetic, and genetic variation analysis of Apicomplexa and Piroplasmids (32, 42–44). It is due to the presence of its multiple copies within the genome (45), a level of sequence conservation, and the existence of hypervariable regions, which result in meaningful phylogenetic comparisons (46, 47). Preliminary studies based on this gene first detected only two clades of *T. equi* in Spain (10) in the year 2004. Soon after, third (genotype C; 2009) and fourth (genotype D; 2010) clades were reported from South Africa (12) and Sudan (15), respectively. Two years later, Qablan et al. (19) reported an additional clade (genotype E) from horses in Jordan (Suwaymah), South Korea and Spain in 2012; thus, giving rise to the contemporary five clades (A–E) of *T. equi* (20–27).

The present study exhibited marked nucleotide variations in the V4 hypervariable region of the 18S rRNA gene, with the presence of four significantly different *T. equi* genotypes (A, B, C and D), in agreement with the results of some of the previous researchers (15–18). Although the majority of sequences (52.85%) of *T. equi* in our dataset accorded to genotype B, our analysis did not firmly support the separate existence of the clades B and E of five genotype classification, in concordance with the key results of Hall et al. (16), and contrary to the findings of Qablan et al. (19). Instead, it indicated integration of both of the former genotypes to generate a distinct clade B of the four clade system, in consonance with the findings of Veronesi et al. (17), Alanazi et al. (48), and Coultous et al. (49). Our results indicated that the genotype B of Nagore et al. (10) and genotype E of Qablan et al. (19) together form the clade B with a high bootstrap value (95%). In addition, *Theileria haneyi* occupied clade C of the current *T. equi* umbrella just like previous studies (50). A change in number of *T. equi* genotypes can affect the diagnostic results, clinical outcome of infection, and therapeutic efficacy. For example, genotype A is reported to be more commonly associated with clinical piroplasmosis than the other genotypes (14). Similarly, repeated treatment with imidocarb dipropionate cleared the single infection of *T. equi* genotype A, but not from horses co-infected with *T. haneyi* (genotype C) and genotype A (51). Nowadays, it is well established that *T. equi* exhibits greater genetic diversity in the 18S rRNA gene as compared to *B. caballi* (32), and *T. equi* isolates diversify even within the same geographical regions (12, 15). It also exhibits a broad host range involving horses (10, 26), domestic donkeys (*Equus asinus*; 13, 17, 23, 27), camels (19), dogs (29, 52, 53), Asiatic wild ass (*Equus hemionus*), African wild donkeys (*Equus africanus*), zebras (*Equus quagga*; 27), and Black rhinoceros (*Diceros bicornis*; 54), which contribute to the maintenance and circulation of the parasite. It also suggests a reduced host specificity of the parasite. *Theileria equi* genotype A has been documented to infect a wider spectrum of hosts, *viz.,* horses, camels, and dogs (19). For *T. equi*, natural recovery is not possible and life-long asymptomatic carriers are seen as opposed to *B. caballi* infection (55). Therefore, a change in the number of genotypes can be expected with an increase in new submissions of *T. equi* sequences in the nucleotide databases from countries/ geographical locations where it has not been reported thus far (21). Furthermore, it is also documented that the endemicity of *T. equi* infection is implicated by only one genotype in combination with (or without) other introduced genotypes in a geographical location (15). Genotype C demonstrated a comparatively higher genetic diversity (91.0–100% identity) contrary to the remaining genotypes. The genotypic variations in the 18S rRNA gene of *T. equi* seems to be due to an increased genetic divergence over a protracted period of time (12, 15, 17). In addition, appearance of single nucleotide polymorphisms (SNPs) in the genome, genetic recombination in the tick vectors, and co-infection of ticks and various hosts with two or more genotypes of *T. equi* within the same population can also be attributed (12). A parallel genetic divergence resulting in generation of similar sequences and/or variations at places other than the site of origin had also been postulated (15). The increased dispersal and gene flow due to migration of various hosts and tick vectors from one geographical area to the other cannot be neglected, as it results in progressive spread of genetic variations to different locations.

The genetic diversity observed in the V4 hypervariable region of this gene of *T. equi* has been in agreement with the earlier reports from Africa (12, 15), Asia (19–21, 25), Europe (10, 14, 17, 26), North America (16), and South America (13, 28). It is pertinent to note that the complete V4 region was not significantly variable; instead, a portion containing 41 molecular signatures between nucleotide positions 113–183 was highly variable between genotypes. It can be targeted for designing primers/ probes for the development of genotype-specific conventional and real-time polymerase chain reaction (PCR) assays. The various implications, *viz.,* taxonomy, virulence, immunological cross-reactivity, infection persistence, diagnosis, and transmission dynamics of the genotypes described here are yet to be studied, particularly in experimental infections, and warrants future research.

The clustering of *T. equi* clades and the length of branches possibly indicate the existence of one or even more new/ cryptic species. However, to confirm this proposition, additional data based on other potential molecular markers need to be generated in addition to a better knowledge of the vectors involved in the transmission (21). Recently, *T. equi* genotype C has been reported to represent a novel species, *T. haneyi*, on the basis of whole genome sequence, and several cryptic *Theileria* species have been collectively classified as *T. equi* (50).

As the 18S rRNA gene sequences originating from different geographical locations were found to group together in the phylogenetic analysis, it can be envisaged that the various genotypes of *T. equi* are not geographically delimited. However, it is reported that genotypes A and B are more prevalent in symptomatic and asymptomatic animals, respectively (14, 16).

Widespread geographical distribution of *T. equi* genotypes was demonstrated worldwide, which was in line with the results of other authors (14, 17, 20, 26, 28). It could be due to movement of tick vectors, and equines for trade and equestrian competitions. Besides, the unrestricted cross-border movement of wild animals cannot be neglected. In spite of that, the underlying reasons for the most restricted geographical distribution of the *T. equi* genotype D amongst all the genotypes are difficult to assert at this moment.

An adequate comparison of the genetic variations between sequences from different countries was carried out in the current study as it involved all the sequences of the V4 hypervariable region available in the GenBank™. Considering the sequence heterogeneity within the V4 hypervariable region of *T. equi* genotypes, the probe based hybridization/ diagnostic techniques should cover the local as well as international strains (including all the four genotypes).

It is important to identify any association between parasite genotypes and tick species, if present. The relationship between genotypes, symptomatology and serological cross-reactivity, as established for canine babesiosis (36), needs to be explored for equine piroplasmosis. For formulating the preventive and control strategies for *T. equi* infection in different countries, the presence and distribution of various genotypes need to be kept in mind. Moreover, studies focusing at possible clinical impact, and concomitant detection of the different *T. equi* genotypes in a single animal and/or tick vector, whose existence cannot be discounted, are necessary. Such investigations would require cloning and sequencing of the PCR amplicons, or additional molecular testing using genotype-specific primers and/or probes.

## 5 Conclusion

The present study indicated the presence of only four previously described *T. equi* genotypes (A, B, C and D) based on the V4 hypervariable region of the 18S rRNA gene. It did not support the independent existence of previously identified genotypes B (10) and E (19). Instead, both these genotypes collectively represent *T. equi* genotype B. The presently identified genetic diversity provides novel insights to the clinicians, researchers, government officials (policy makers) and animal owners for the control of equine piroplasmosis caused by *T. equi* in domestic and wild animals.

## Data availability statement

The datasets presented in this study can be found in online repositories. The names of the repository/repositories and accession number(s) can be found in the article/Supplementary material.

## Author contributions

AN: Conceptualization, Formal analysis, Software, Supervision, Writing – review & editing. AK: Formal analysis, Investigation, Methodology, Software, Writing – original draft. AM: Investigation, Methodology, Writing – review & editing. SV: Investigation, Supervision, Writing – review & editing.

## Supplementary material

Supplementary Figure S1 A maximum likelihood tree based on the V4 hypervariable region of the 736 *T. equi* and six *T. haneyi* sequences clearly depicts the four genotypes/clades (A, B, C, and D) of *T. equi* due to extensive nucleotide heterogeneity in this region. The taxon name of each sequence is depicted by its accession number followed by the country of origin.

## References

[1] Schein E. Equine babesiosis In: Ristic M, editor. Babesiosis of domestic animals and man. CRC Press: Boca Raton, FL (1988). 197–208.

[2] Mehlhorn H Schein E. Redescription of *Babesia equi* Laveran, 1901 as *Theileria equi* Mehlhorn, Schein 1998. Parasitol Res. (1998) 84:467–75. doi: 10.1007/s004360050431, PMID: 9660136

[3] Knowles D Jr. Equine babesiosis (piroplasmosis): a problem in the international movement of horses. Br Vet J. (1996) 152:123–6. doi: 10.1016/S0007-1935(96)80066-2, PMID: 8680834

[4] Wise LN Pelzel-McCluskey AM Mealey RH Knowles DP. Equine piroplasmosis. Vet Clin North Am Equine Pract. (2014) 30:677–93. doi: 10.1016/j.cveq.2014.08.008 25300637

[5] Allsopp MTEP Lewis BD Penzhorn BL. Molecular evidence for transplacental transmission of *Theileria equi* from carrier mares to their apparently healthy foals. Vet Parasitol. (2007) 148:130–6. doi: 10.1016/j.vetpar.2007.05.017, PMID: 17601669

[6] Georges KC Ezeokoli CD Sparagano O Pargass I Campbell M D’Abadie R. A case of transplacental transmission of *Theileria equi* in a foal in Trinidad. Vet Parasitol. (2011) 175:363–6. doi: 10.1016/j.vetpar.2010.10.019, PMID: 21051152

[7] de Waal DT. Equine piroplasmosis: a review. Br Vet J. (1992) 148:6–14. doi: 10.1016/0007-1935(92)90061-5 1551016

[8] Zobba R Ardu M Niccolini S Chessa B Manna L Cocco R. Clinical and laboratory findings in equine piroplasmosis. J Equine Vet. (2008) 28:301–8. doi: 10.1016/j.jevs.2008.03.005

[9] Wise LN Kappmeyer LS Mealey RH Knowles DP. Review of equine piroplasmosis. J Vet Intern Med. (2013) 27:1334–46. doi: 10.1111/jvim.12168, PMID: 24033559

[10] Nagore D García-Sanmartín J García-Pérez AL Juste RA Hurtado A. Detection and identification of equine *Theileria* and *Babesia* species by reverse line blotting: epidemiological survey and phylogenetic analysis. Vet Parasitol. (2004) 123:41–54. doi: 10.1016/j.vetpar.2004.04.010, PMID: 15265570

[11] Kouam MK Kantzoura V Masuoka PM Gajadhar AA Theodoropoulos G. Genetic diversity of equine piroplasms in Greece with a note on speciation within *Theileria* genotypes (*T. Equi* and *T. Equi*-like). Infect Genet Evol. (2010) 10:963–8. doi: 10.1016/j.meegid.2010.06.008 20601168

[12] Bhoora R Franssen L Oosthuizen MC Guthrie AJ Zweygarth E Penzhorn BL. Sequence heterogeneity in the 18S rRNA gene within *Theileria equi* and *Babesia caballi* from horses in South Africa. Vet Parasitol. (2009) 159:112–20. doi: 10.1016/j.vetpar.2008.10.004, PMID: 19019541

[13] Braga MDSCDO Costa FN Gomes DRM Xavier DR André MR Gonçalves LR. Genetic diversity of piroplasmids species in equids from island of São Luís, northeastern Brazil. Rev Bras Parasitol Vet. (2017) 26:331–9. doi: 10.1590/s1984-29612017046, PMID: 28977247

[14] Manna G Cersini A Nardini R Bartolome Del Pino LE Antognetti V Zini M. Genetic diversity of *Theileria equi* and *Babesia caballi* infecting horses of central-southern Italy and preliminary results of its correlation with clinical and serological status. Ticks Tick Borne Dis. (2018) 9:1212–20. doi: 10.1016/j.ttbdis.2018.05.005, PMID: 29752142

[15] Salim B Bakheit MA Kamau J Nakamura I Sugimoto C. Nucleotide sequence heterogeneity in the small subunit ribosomal RNA gene within *Theileria equi* from horses in Sudan. Parasitol Res. (2010) 106:493–8. doi: 10.1007/s00436-009-1691-7, PMID: 19953269

[16] Hall CM Busch JD Scoles GA Palma-Cagle KA Ueti MW Kappmeyer LS. Genetic characterization of Theileria equi infecting horses in North America: evidence for a limited source of U.S. introductions. Parasit Vectors. (2013) 6:1–12. doi: 10.1186/1756-3305-6-35 23399005 PMC3606381

[17] Veronesi F Morganti G Ravagnan S Laus F Spaterna A Diaferia M. Molecular and serological detection of tick-borne pathogens in donkeys (*Equus asinus*) in Italy. Vet Microbiol. (2014) 173:348–54. doi: 10.1016/j.vetmic.2014.08.017, PMID: 25213231

[18] Torres R Hurtado C Pérez-Macchi S Bittencourt P Freschi C de Melo VVC. Occurrence and genetic diversity of *Babesia caballi* and *Theileria equi* in Chilean thoroughbred racing horses. Pathogens. (2021) 10:714. doi: 10.3390/pathogens10060714, PMID: 34200433 PMC8226895

[19] Qablan MA Sloboda M Jirků M Oborník M Dwairi S Amr ZS. Quest for the piroplasms in camels: identification of *Theileria equi* and *Babesia caballi* in Jordanian dromedaries by PCR. Vet Parasitol. (2012) 186:456–60. doi: 10.1016/j.vetpar.2011.11.070, PMID: 22186193

[20] Munkhjargal T Sivakumar T Battsetseg B Nyamjargal T Aboulaila M Purevtseren B. Prevalence and genetic diversity of equine piroplasms in Tov province, Mongolia. Infect Genet Evol. (2013) 16:178–85. doi: 10.1016/j.meegid.2013.02.005, PMID: 23416256

[21] Qablan MA Obornik M Petrzelkova KJ Sloboda M Shudiefat MF Horin P. Infections by *Babesia caballi* and *Theileria equi* in Jordanian equids: epidemiology and genetic diversity. Parasitology. (2013) 140:1096–103. doi: 10.1017/S0031182013000486, PMID: 23673249

[22] Liu Q Meli ML Zhang Y Meili T Stirn M Riond B. Sequence heterogeneity in the 18S rRNA gene in *Theileria equi* from horses presented in Switzerland. Vet Parasitol. (2016) 221:24–9. doi: 10.1016/j.vetpar.2016.03.003, PMID: 27084467

[23] Ozubek S Aktas M. Genetic diversity and prevalence of piroplasm species in equids from Turkey. Comp Immunol Microbiol Infect Dis. (2018) 59:47–51. doi: 10.1016/j.cimid.2018.08.005 30290887

[24] Peckle M Pires MS da Silva CB da Costa RL Vitari GLV Senra MVX. Molecular characterization of *Theileria equi* in horses from the state of Rio de Janeiro, Brazil. Ticks Tick Borne Dis. (2018) 9:349–53. doi: 10.1016/j.ttbdis.2017.11.011, PMID: 29223587

[25] Wang J Liu J Yang J Wang X Li Z Jianlin X. The first molecular detection and genetic diversity of *Babesia caballi* and *Theileria equi* in horses of Gansu province, China. Ticks Tick Borne Dis. (2019) 10:528–32. doi: 10.1016/j.ttbdis.2019.01.003, PMID: 30670354

[26] Camino E Cruz-Lopez F de Juan L Dominguez L Shiels B Coultous RM. Phylogenetic analysis and geographical distribution of *Theileria equi* and *Babesia caballi* sequences from horses residing in Spain. Ticks Tick Borne Dis. (2020) 11:101521. doi: 10.1016/j.ttbdis.2020.101521, PMID: 32993941

[27] Tirosh-Levy S Gottlieb Y Arieli O Mazuz ML King R Horowitz I. Genetic characteristics of *Theileria equi* in zebras, wild and domestic donkeys in Israel and the Palestinian authority. Ticks Tick Borne Dis. (2020) 11:101286. doi: 10.1016/j.ttbdis.2019.101286, PMID: 31537490

[28] de Sousa KCM Fernandes MP Herrera HM Freschi CR Machado RZ André MR. Diversity of piroplasmids among wild and domestic mammals and ectoparasites in Pantanal wetland, Brazil. Ticks Tick Borne Dis. (2018) 9:245–53. doi: 10.1016/j.ttbdis.2017.09.010, PMID: 28941935

[29] Beck R Vojta L Mrljak V Marinculić A Beck A Živičnjak T. Diversity of *Babesia* and *Theileria* species in symptomatic and asymptomatic dogs in Croatia. Int J Parasitol. (2009) 39:843–8. doi: 10.1016/j.ijpara.2008.12.005, PMID: 19367832

[30] Criado-Fornelio A Gónzalez-del-Rıo MA Buling-Saraña A Barba-Carretero JC. The “expanding universe” of piroplasms. Vet Parasitol. (2004) 119:337–45. doi: 10.1016/j.vetpar.2003.11.015, PMID: 15154598

[31] Ros-García A M’ghirbi Y Hurtado A Bouattour A. Prevalence and genetic diversity of piroplasm species in horses and ticks from Tunisia. Infect Genet Evol. (2013) 17:33–7. doi: 10.1016/j.meegid.2013.03.038, PMID: 23542456

[32] Nehra AK Kumari A Moudgil AD Vohra S. Phylogenetic analysis, genetic diversity and geographical distribution of *Babesia caballi* based on 18S rRNA gene. Ticks Tick Borne Dis. (2021) 12:101776. doi: 10.1016/j.ttbdis.2021.101776, PMID: 34271342

[33] Kumar S Stecher G Li M Knyaz C Tamura K. MEGA X: molecular evolutionary genetics analysis across computing platforms. Mol Biol Evol. (2018) 35:1547–9. doi: 10.1093/molbev/msy096, PMID: 29722887 PMC5967553

[34] Thompson JD Higgins DG Gibson TJ. CLUSTAL W: improving the sensitivity of progressive multiple sequence alignment through sequence weighting, position specific gap penalties and weight matrix choice. Nucleic Acids Res. (1994) 22:4673–80. doi: 10.1093/nar/22.22.4673, PMID: 7984417 PMC308517

[35] Katoh K Rozewicki J Yamada KD. MAFFT online service: multiple sequence alignment, interactive sequence choice and visualization. Brief Bioinform. (2019) 20:1160–6. doi: 10.1093/bib/bbx108, PMID: 28968734 PMC6781576

[36] Kimura MA. A simple method for estimating evolutionary rates of base substitutions through comparative studies of nucleotide sequences.. J Mol Evol. (1980) 16:111–20. doi: 10.1007/BF01731581, PMID: 7463489

[37] Battsetseg B Lucero S Xuan X Claveria FG Inoue N Alhassan A. Detection of natural infection of *Boophilus microplus* with *Babesia equi* and *Babesia caballi* in Brazilian horses using nested polymerase chain reaction. Vet Parasitol. (2002) 107:351–7. doi: 10.1016/S0304-4017(02)00131-0, PMID: 12163246

[38] Díaz-Sánchez AA Pires MS Estrada CY Cañizares EV del Castillo Domínguez SL Cabezas-Cruz A. First molecular evidence of *Babesia caballi* and *Theileria equi* infections in horses in Cuba. Parasitol Res. (2018) 117:3109–18. doi: 10.1007/s00436-018-6005-5, PMID: 30033488

[39] Sunday Idoko I Tirosh-Levy S Leszkowicz Mazuz M Mohammed Adam B Sikiti Garba B Wesley Nafarnda D. Genetic characterization of piroplasms in donkeys and horses from Nigeria. Animals. (2020) 10:324. doi: 10.3390/ani10020324, PMID: 32085574 PMC7070495

[40] Wu J Cui Y Yu F Muhatai G Tao D Zhao A. Prevalence and genetic characterization of *Theileria equi* and *Babesia caballi* in grazing horses in Xinjiang, northwestern China. Parasitol Res. (2023) 122:387–94. doi: 10.1007/s00436-022-07749-4, PMID: 36504396

[41] Cacciò S Cammà C Onuma M Severini C. The β-tubulin gene of *Babesia* and *Theileria* parasites is an informative marker for species discrimination. Int J Parasitol. (2000) 30:1181–5. doi: 10.1016/S0020-7519(00)00105-3, PMID: 11027785

[42] Morrison DA. Evolution of the Apicomplexa: where are we now? Trends Parasitol. (2009) 25:375–82. doi: 10.1016/j.pt.2009.05.010 19635681

[43] Lack JB Reichard MV Van Den Bussche RA. Phylogeny and evolution of the Piroplasmida as inferred from 18S rRNA sequences. Int J Parasitol. (2012) 42:353–63. doi: 10.1016/j.ijpara.2012.02.005, PMID: 22429769

[44] Chauhan RP Kumari A Nehra AK Ram H Garg R Banerjee PS. Genetic characterization and phylogenetic analysis of *Sarcocystis suihominis* infecting domestic pigs (*Sus scrofa*) in India. Parasitol Res. (2020) 119:3347–57. doi: 10.1007/s00436-020-06857-3, PMID: 32833051

[45] Hunfeld KP Hildebrandt A Gray JS. Babesiosis: recent insights into an ancient disease. Int J Parasitol. (2008) 38:1219–37. doi: 10.1016/j.ijpara.2008.03.001, PMID: 18440005

[46] Katzer F McKellar S Kirvar E Shiels B. Phylogenetic analysis of *Theileria* and *Babesia equi* in relation to the establishment of parasite populations within novel host species and the development of diagnostic tests. Mol Biochem Parasitol. (1998) 95:33–44. doi: 10.1016/S0166-6851(98)00085-1, PMID: 9763287

[47] Allsopp MTEP Allsopp BA. Molecular sequence evidence for the reclassification of SomeBabesiaSpecies. Ann N Y Acad Sci. (2006) 1081:509–17. doi: 10.1196/annals.1373.076 17135560

[48] Alanazi AD Said AE Morin-Adeline V Alyousif MS Slapeta J. Quantitative PCR detection of *Theileria equi* using laboratory workflows to detect asymptomatic persistently infected horses. Vet Parasitol. (2014) 206:138–45. doi: 10.1016/j.vetpar.2014.09.019, PMID: 25450724

[49] Coultous RM McDonald M Raftery AG Shiels BR Sutton DGM Weir W. Analysis of *Theileria equi* diversity in the Gambia using a novel genotyping method. Transbound Emerg Dis. (2019) 67:1213–21. doi: 10.1111/tbed.13454 31845493

[50] Knowles DP Kappmeyer LS Haney D Herndon DR Fry LM Munro JB. Discovery of a novel species, *Theileria haneyi* n. sp., infective to equids, highlights exceptional genomic diversity within the genus *Theileria*: implications for apicomplexan parasite surveillance. Int J Parasitol. (2018) 48:679–90. doi: 10.1016/j.ijpara.2018.03.010, PMID: 29885436

[51] Sears K Knowles D Dinkel K Mshelia PW Onzere C Silva M. Imidocarb dipropionate lacks efficacy against *Theileria haneyi* and fails to consistently clear *Theileria equi* in horses co-infected with *T*. *Haneyi*. Pathogens. (2020) 9:1035. doi: 10.3390/pathogens9121035, PMID: 33321715 PMC7764667

[52] Criado-Fornelio A Martinez-Marcos A Buling-Sarana A Barba-Carretero JC. Molecular studies on *Babesia*, *Theileria* and *Hepatozoon* in southern Europe. Part I. epizootiological aspects. Vet Parasitol. (2003) 113:189–201. doi: 10.1016/S0304-4017(03)00078-5, PMID: 12719133

[53] Qablan MA Kubelová M Široký P Modrý D Amr ZS. Stray dogs of northern Jordan as reservoirs of ticks and tick-borne hemopathogens. Parasitol Res. (2012) 111:301–7. doi: 10.1007/s00436-012-2839-4, PMID: 22434363

[54] Zimmermann DE Penzhorn BL Vorster I Troskie M Oosthuizen MC. *Babesia bicornis*, *Theileria bicornis* and *Theileria equi* in metapopulations of two black rhinoceros (*Diceros bicornis*) subspecies in South Africa and their potential impact on conservation. Ticks Tick Borne Dis. (2021) 12:101635. doi: 10.1016/j.ttbdis.2020.101635, PMID: 33373893

[55] Bruning A. Equine piroplasmosis an update on diagnosis, treatment and prevention. Br Vet J. (1996) 152:139–51. doi: 10.1016/S0007-1935(96)80070-4, PMID: 8680838

