# Supplementary figures and images for: Revisiting the genotypes of Theileria equi based on the V4 hypervariable region of the 18S rRNA gene
Source: Front Vet Sci. 2024 Mar 15;11:1303090. doi: 10.3389/fvets.2024.1303090 (PMC10978764; doi:10.3389/fvets.2024.1303090)

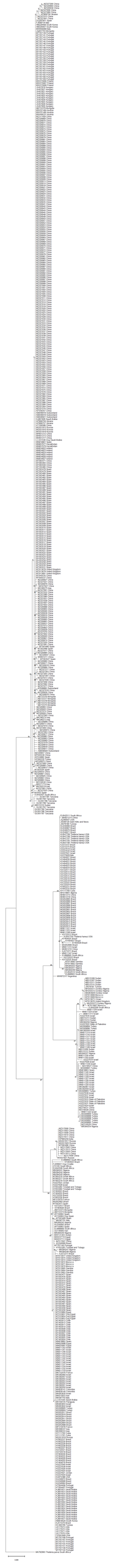

Supplement: Supplementary Figure S1 — A maximum likelihood tree based on the V4 hypervariable region of the 736 T. equi and six T. haneyi sequences clearly depicts the four genotypes/clades (A, B, C, and D) of T. equi due to extensive nucleotide heterogeneity in this region. The taxon name of each sequence is depicted by its accession number followed by the country of origin. [file Image_1.PNG]
